# Steviamine, a new class of indolizidine alkaloid [(1*R*,2*S*,3*R*,5*R*,8a*R*)-3-hydroxy­meth­yl-5-methyl­octa­hydro­indolizine-1,2-diol hydro­bromide]

**DOI:** 10.1107/S1600536809043827

**Published:** 2009-10-28

**Authors:** Amber L. Thompson, Agnieszka Michalik, Robert J. Nash, Francis X. Wilson, Renate van Well, Peter Johnson, George W. J. Fleet, Chu-Yi Yu, Xiang-Guo Hu, Richard I. Cooper, David J. Watkin

**Affiliations:** a Chemical Crystallography, Inorganic Chemistry Laboratory, South Parks Road, Oxford OX1 3QR, England; b Phytoquest Limited, IBERS, Plas Gogerddan, Aberystwyth SY23 3EB, Ceredigion, Wales; c Summit PLC, 91, Milton Park, Abingdon, Oxfordshire OX14 4RY, England; d Chemistry Research Laboratory, University of Oxford, Mansfield Road, Oxford, OX1 3TA, England; e Institute of Chemistry, Chinese Academy of Science, Beijing 100190, People’s Republic of China

## Abstract

X-ray crystallographic analysis of the title hydro­bromide salt, C_10_H_20_N^+^·Br^−^, of (1*R*,2*S*,3*R*,5*R*,8a*R*)-3-hydroxy­meth­yl-5-methyl­octa­hydro­indolizine-1,2-diol defines the absolute and relative stereochemistry at the five chiral centres in steviamine, a new class of polyhydroxy­lated indolizidine alkaloid isolated from *Stevia rebaudiana* (Asteraceae) leaves. In the crystal structure, mol­ecules are linked by inter­molecular O—H⋯Br and N—H⋯Br hydrogen bonds, forming double chains around the twofold screw axes along the *b*-axis direction. Intra­molecular O—H⋯O inter­actions occur.

## Related literature

For background to the biological activity of indolizidines, see: Asano *et al.* (2000*a* ▶,2000*b* ▶); Colegate *et al.* (1979 ▶); Davis *et al.* (1996 ▶); Donohoe *et al.* (2008 ▶); Durantel (2009 ▶); Hakansson *et al.* (2008 ▶); Hohenschutz *et al.* (1981 ▶); Kato *et al.* (1999 ▶, 2007 ▶); Klein *et al.* (1999 ▶); Lagana *et al.* (2006 ▶); Sengoku *et al.* (2009 ▶); Watson *et al.* (2001 ▶); Whitby *et al.* (2005 ▶); Yamashita *et al.* (2002 ▶). For the Hooft parameter, see: Hooft *et al.* (2008 ▶). For the extinction correction, see: Larson (1970 ▶).
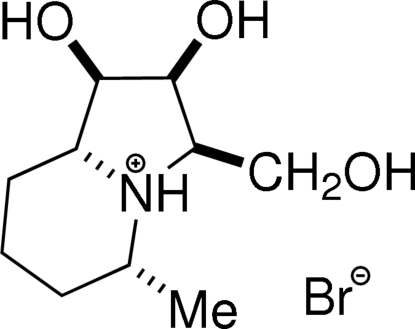


## Experimental

### 

#### Crystal data

C_10_H_20_N^+^·Br^−^
*M* *_r_* = 282.18
Orthorhombic, 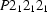

*a* = 8.4616 (1) Å
*b* = 8.8762 (1) Å
*c* = 15.8270 (2) Å
*V* = 1188.72 (2) Å^3^
*Z* = 4
Mo *K*α radiation
μ = 3.45 mm^−1^
*T* = 150 K
0.46 × 0.46 × 0.26 mm

#### Data collection

Nonius KappaCCD area-detector diffractometer
Absorption correction: multi-scan (*DENZO*/*SCALEPACK*; Otwinowski & Minor, 1997 ▶) *T*_min_ = 0.20, *T*_max_ = 0.41
2675 measured reflections
2658 independent reflections
2484 reflections with *I* > 2σ(*I*)
*R*_int_ = 0.042

#### Refinement

*R*[*F* ^2^ > 2σ(*F* ^2^)] = 0.025
*wR*(*F* ^2^) = 0.052
*S* = 1.00
2658 reflections
138 parameters
H-atom parameters constrained
Δρ_max_ = 0.37 e Å^−3^
Δρ_min_ = −0.52 e Å^−3^
Absolute structure: Flack (1983 ▶), 1102 Friedel pairs
Flack parameter: 0.002 (10)

### 

Data collection: *COLLECT* (Nonius, 2001 ▶).; cell refinement: *DENZO*/*SCALEPACK* (Otwinowski & Minor, 1997 ▶); data reduction: *DENZO*/*SCALEPACK*; program(s) used to solve structure: *SIR92* (Altomare *et al.*, 1994 ▶); program(s) used to refine structure: *CRYSTALS* (Betteridge *et al.*, 2003 ▶); molecular graphics: *CAMERON* (Watkin *et al.*, 1996 ▶); software used to prepare material for publication: *CRYSTALS*.

## Supplementary Material

Crystal structure: contains datablocks I, global. DOI: 10.1107/S1600536809043827/lh2918sup1.cif

Structure factors: contains datablocks I. DOI: 10.1107/S1600536809043827/lh2918Isup2.hkl

Additional supplementary materials:  crystallographic information; 3D view; checkCIF report

## Figures and Tables

**Table 1:** Hydrogen-bond geometry (Å, °)

*D*—H⋯*A*	*D*—H	H⋯*A*	*D*⋯*A*	*D*—H⋯*A*
O5—H51⋯O2	0.84	2.34	2.684 (3)	105
O5—H51⋯O15	0.84	2.53	3.018 (3)	118
N7—H71⋯Br1	0.98	2.29	3.268 (2)	172
O2—H21⋯Br1^i^	0.82	2.55	3.364 (2)	177
O15—H151⋯Br1^ii^	0.84	2.39	3.211 (2)	169

Symmetry codes: (i) 
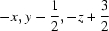
; (ii) 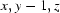.

**Fig. 1.**
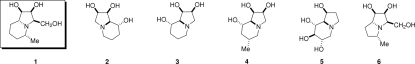
Chemical structures of compounds 1 - 6.

**Fig. 2.**
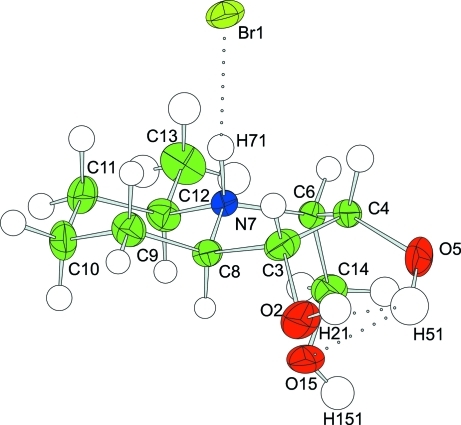
The molecular structure of the title compound with displacement ellipsoids drawn at the 50% probability level. Hydrogen bonds are shown with a dotted lines.

**Fig. 3.**
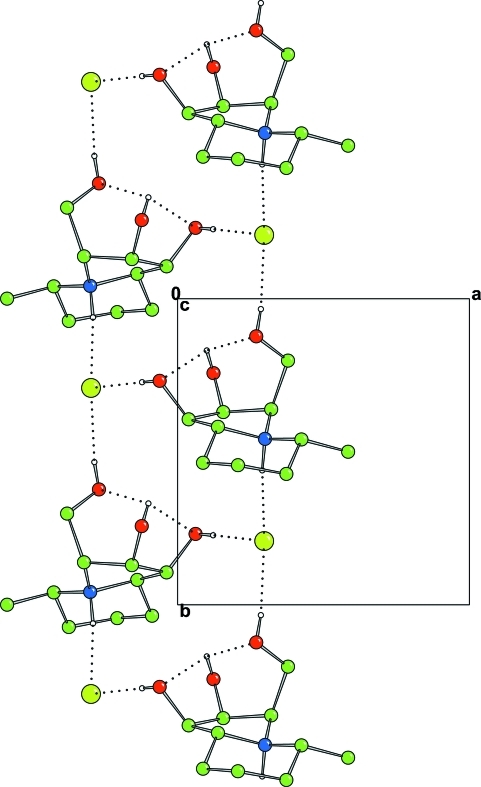
1 forms hydrogen bonded double chains around around the twofold screw axis parallel to the b-axis (viewed down the c-axis). Hydrogen bonding interactions are shown as dotted lines and all hydrogen atoms not involved are omitted for clarity.

## supplementary crystallographic information

### Comment

Well over 100 iminosugars - analogues of sugars in which the ring oxygen is replaced by nitrogen - have been isolated as natural products (Asano *et al.*, 2000*a*; Watson *et al.*, 2001). This paper establishes both the relative and absolute stereochemistry of the five chiral centres in steviamine (1), recently isolated from the leaves of Stevia rebaudiana (Asteraceae); (1) is the first example of a new class of indolizidine alkaloid with an alkyl group attached to the piperidine ring. Swainsonine (2, see Fig. 1), a trihydroxyindolizidine isolated from Swainsona canescens (Colegate *et al.*, 1979), is a powerful inhibitor of α-mannosidases and has potential as a chemotherapeutic agent for the treatment of cancer (Lagana *et al.*, 2006; Klein *et al.*, 1999). l-Swainsonine 3, the enantiomer of 2, is a very powerful α-rhamnosidase inhibitor (Davis *et al.*, 1996); 4 in which a methyl group is introduced into the piperidine ring is nearly 100 times more potent an inhibitor than 2 (Hakansson *et al.*, 2008). Castanospermine 5, isolated from Castanospermum australe (Hohenschutz *et al.*, 1981), is an inhibitor of some α-glucosidases and a potent inhibitor of dengue virus infection *in vivo* (Whitby *et al.*, 2005); Celgosivir, a simple derivative of 5, is in development for the treatment of HCV infection (Durantel, 2009). Hyacinthacine A4 6, isolated from Scilla sibirica (Asano *et al.*, 2000*b*; Yamashita *et al.*, 2002), is the pyrrolizidine equivalent of steviamine 1. Many hyacinthacines have been isolated from a range of plants (Kato *et al.*, 1999; Kato *et al.*, 2007) and have attracted considerable attention from synthetic organic chemists (Sengoku *et al.*, 2009; Donohoe *et al.*, 2008). Steviamine 1 is unlikely to be the only naturally occurring indolizidine with a methyl branch which will provide similarly challenging synthetic targets.

As a natural product, the crystal was expected to be enantiopure and the Flack *x* parameter refined to 0.002 (10) (Flack, 1983). Analysis of the Bijvoet differences using within *CRYSTALS* (Betteridge *et al.*, 2003) gives the Hooft *y* parameter as 0.023 (6), indicating that the probability that the configuration is incorrect allowing for the posibility of racemic twinning is less than 0.000001% (Hooft *et al.*, 2008).

On examination of hydrogen bonding interactions in 1, the position of H51 initially seemed incorrect, lying between atoms O2 and O15. However, examination of the difference map indicates the presence of a peak believed to be a hydrogen atom which moves little on refinement suggesting the hydrogen bond is bifurcated (Fig. 2, Table 1). The molecules are linked together by three hydrogen bonds (two O—H···Br and one N—H···Br, Table 1) to form double chains around the twofold screw axes along the *b* direction (Fig. 3).

### Experimental

Steviamine was isolated by a combination of strongly acidic cation, and strongly basic anion, exchange chromatography. The compound was retained on cation exchange resin (IR120) and was chromatographed on the anion exchange resin (CG400) from which it was eluted with water. Isolation was monitored using GC-MS of the trimethylsilyl-derivative (distinctive major ion at 314 amu). Steviamine was crystallized as its hydrobromide salt from ethanol.

### Refinement

The H atoms were all located in a difference map, but those attached to carbon atoms were repositioned geometrically. The H atoms were initially refined separately with soft restraints on the bond lengths and angles to regularize their geometry (C—H in the range 0.93–0.98, O—H = 0.82 Å) and *U*_iso_(H) (in the range 1.2–1.5 times *U*_eq_ of the parent atom), after which the positions were refined with riding constraints.

On comparison of *F*_o_ and *F*_c_, it was apparent that for large values, of *F*_o_ was noticably less than *F*_c_, so an extinction parameter was refined (Larson, 1970).

### Crystal data

**Table:**

C_10_H_20_N^+^·Br^−^	*D*_x_ = 1.577 Mg m^−^^3^
*M**_r_* = 282.18	Melting point: not measured K
Orthorhombic, *P*2_1_2_1_2_1_	Mo *K*α radiation, λ = 0.71073 Å
Hall symbol: P 2ac 2ab	Cell parameters from 1544 reflections
*a* = 8.4616 (1) Å	θ = 5–27°
*b* = 8.8762 (1) Å	µ = 3.45 mm^−^^1^
*c* = 15.8270 (2) Å	*T* = 150 K
*V* = 1188.72 (2) Å^3^	Plate, colourless
*Z* = 4	0.46 × 0.46 × 0.26 mm
*F*(000) = 584	

### Data collection

**Table:**

Nonius KappaCCD area-detector diffractometer	2484 reflections with *I* > 2σ(*I*)
graphite	*R*_int_ = 0.042
ω scans	θ_max_ = 27.5°, θ_min_ = 5.1°
Absorption correction: multi-scan (*DENZO*/*SCALEPACK*; Otwinowski & Minor, 1997)	*h* = −10→10
*T*_min_ = 0.20, *T*_max_ = 0.41	*k* = −11→11
2675 measured reflections	*l* = −20→20
2658 independent reflections	

### Refinement

**Table:**

Refinement on *F*^2^	H-atom parameters constrained
Least-squares matrix: full	Method = Modified Sheldrick *w* = 1/[σ^2^(*F*^2^) + ( 0.01*P*)^2^ + 0.86*P*], where *P* = [max(*F*_o_^2^,0) + 2*F*_c_^2^]/3
*R*[*F*^2^ > 2σ(*F*^2^)] = 0.025	(Δ/σ)_max_ = 0.001
*wR*(*F*^2^) = 0.052	Δρ_max_ = 0.37 e Å^−^^3^
*S* = 1.00	Δρ_min_ = −0.52 e Å^−^^3^
2658 reflections	Extinction correction: Larson (1970), Equation 22
138 parameters	Extinction coefficient: 75 (8)
0 restraints	Absolute structure: Flack (1983), 1102 Friedel-pairs
Primary atom site location: structure-invariant direct methods	Flack parameter: 0.002 (10)
Hydrogen site location: inferred from neighbouring sites	

### Fractional atomic coordinates and isotropic or equivalent isotropic displacement parameters (Å^2^)

**Table:**

	*x*	*y*	*z*	*U*_iso_*/*U*_eq_	
Br1	0.29652 (3)	0.79140 (2)	0.693045 (16)	0.0373	
O2	−0.0613 (2)	0.2681 (2)	0.63610 (12)	0.0470	
C3	0.0377 (3)	0.3939 (3)	0.65007 (17)	0.0381	
C4	0.1564 (4)	0.3703 (3)	0.72343 (15)	0.0436	
O5	0.1230 (3)	0.2427 (2)	0.77338 (12)	0.0682	
C6	0.3207 (4)	0.3611 (2)	0.68353 (14)	0.0362	
N7	0.2994 (3)	0.45785 (19)	0.60566 (10)	0.0248	
C8	0.1381 (3)	0.4183 (3)	0.57125 (14)	0.0267	
C9	0.0873 (3)	0.5356 (3)	0.50829 (16)	0.0349	
C10	0.2069 (4)	0.5435 (3)	0.43633 (14)	0.0389	
C11	0.3726 (3)	0.5724 (3)	0.47093 (16)	0.0394	
C12	0.4244 (3)	0.4594 (3)	0.53787 (15)	0.0330	
C13	0.5841 (3)	0.4996 (4)	0.5756 (2)	0.0490	
C14	0.3784 (4)	0.2019 (3)	0.66419 (17)	0.0517	
O15	0.2695 (3)	0.12282 (19)	0.61308 (12)	0.0522	
H31	−0.0258	0.4840	0.6613	0.0459*	
H41	0.1545	0.4576	0.7609	0.0522*	
H61	0.4003	0.4116	0.7186	0.0435*	
H81	0.1481	0.3216	0.5435	0.0325*	
H92	0.0822	0.6334	0.5366	0.0421*	
H91	−0.0170	0.5096	0.4861	0.0419*	
H102	0.1762	0.6219	0.3973	0.0473*	
H101	0.2044	0.4458	0.4063	0.0468*	
H111	0.3741	0.6720	0.4958	0.0478*	
H112	0.4450	0.5700	0.4237	0.0485*	
H121	0.4243	0.3580	0.5138	0.0384*	
H132	0.6602	0.5024	0.5310	0.0746*	
H131	0.5781	0.5970	0.6021	0.0735*	
H133	0.6131	0.4254	0.6167	0.0738*	
H141	0.3923	0.1509	0.7163	0.0614*	
H142	0.4798	0.2080	0.6355	0.0607*	
H151	0.2850	0.0336	0.6278	0.0778*	
H21	−0.1216	0.2726	0.6762	0.0723*	
H51	0.0999	0.1687	0.7424	0.1018*	
H71	0.2900	0.5607	0.6279	0.0500*	

### Atomic displacement parameters (Å^2^)

**Table:**

	*U*^11^	*U*^22^	*U*^33^	*U*^12^	*U*^13^	*U*^23^
Br1	0.04557 (13)	0.02142 (11)	0.04505 (13)	−0.00181 (11)	0.00573 (13)	−0.00477 (10)
O2	0.0451 (11)	0.0463 (11)	0.0495 (11)	−0.0168 (9)	0.0134 (8)	0.0013 (9)
C3	0.0472 (16)	0.0264 (12)	0.0406 (14)	−0.0030 (11)	0.0188 (12)	−0.0031 (10)
C4	0.078 (2)	0.0311 (12)	0.0220 (11)	−0.0217 (13)	0.0128 (12)	−0.0036 (9)
O5	0.1153 (19)	0.0591 (14)	0.0301 (9)	−0.0488 (13)	−0.0052 (11)	0.0145 (9)
C6	0.0647 (17)	0.0209 (10)	0.0230 (11)	−0.0009 (11)	−0.0096 (13)	0.0022 (9)
N7	0.0324 (9)	0.0199 (8)	0.0220 (8)	−0.0004 (9)	0.0005 (9)	−0.0002 (6)
C8	0.0307 (12)	0.0229 (10)	0.0266 (11)	−0.0031 (9)	0.0027 (10)	−0.0016 (8)
C9	0.0349 (13)	0.0358 (13)	0.0339 (13)	0.0056 (11)	−0.0047 (11)	−0.0005 (11)
C10	0.0489 (14)	0.0414 (13)	0.0263 (11)	0.0028 (14)	−0.0021 (13)	0.0093 (10)
C11	0.0466 (15)	0.0391 (14)	0.0326 (13)	−0.0071 (12)	0.0113 (12)	0.0065 (11)
C12	0.0317 (13)	0.0356 (13)	0.0316 (12)	0.0015 (10)	0.0044 (10)	−0.0048 (10)
C13	0.0316 (14)	0.0627 (19)	0.0526 (17)	0.0008 (13)	0.0022 (13)	−0.0101 (14)
C14	0.091 (2)	0.0238 (12)	0.0401 (13)	0.0082 (16)	−0.0209 (14)	−0.0002 (12)
O15	0.0927 (18)	0.0219 (8)	0.0419 (10)	0.0030 (10)	−0.0184 (11)	−0.0038 (7)

### Geometric parameters (Å, °)

**Table:**

O2—C3	1.414 (3)	C9—H92	0.978
O2—H21	0.815	C9—H91	0.977
C3—C4	1.549 (4)	C10—C11	1.527 (4)
C3—C8	1.525 (3)	C10—H102	0.965
C3—H31	0.980	C10—H101	0.989
C4—O5	1.409 (3)	C11—C12	1.523 (4)
C4—C6	1.529 (4)	C11—H111	0.968
C4—H41	0.976	C11—H112	0.967
O5—H51	0.843	C12—C13	1.519 (4)
C6—N7	1.513 (3)	C12—H121	0.977
C6—C14	1.526 (3)	C13—H132	0.955
C6—H61	0.981	C13—H131	0.963
N7—C8	1.511 (3)	C13—H133	0.958
N7—C12	1.507 (3)	C14—O15	1.412 (3)
N7—H71	0.982	C14—H141	0.949
C8—C9	1.504 (3)	C14—H142	0.972
C8—H81	0.967	O15—H151	0.836
C9—C10	1.525 (4)		
			
C3—O2—H21	102.1	C8—C9—H91	109.4
O2—C3—C4	113.2 (2)	C10—C9—H91	110.0
O2—C3—C8	108.3 (2)	H92—C9—H91	109.5
C4—C3—C8	105.7 (2)	C9—C10—C11	110.44 (19)
O2—C3—H31	110.4	C9—C10—H102	109.4
C4—C3—H31	109.3	C11—C10—H102	110.8
C8—C3—H31	109.8	C9—C10—H101	107.7
C3—C4—O5	113.5 (2)	C11—C10—H101	109.8
C3—C4—C6	106.69 (19)	H102—C10—H101	108.6
O5—C4—C6	111.8 (2)	C10—C11—C12	113.8 (2)
C3—C4—H41	109.7	C10—C11—H111	108.1
O5—C4—H41	107.1	C12—C11—H111	108.4
C6—C4—H41	107.9	C10—C11—H112	107.5
C4—O5—H51	110.3	C12—C11—H112	109.9
C4—C6—N7	101.4 (2)	H111—C11—H112	109.0
C4—C6—C14	115.0 (2)	C11—C12—N7	107.4 (2)
N7—C6—C14	113.58 (19)	C11—C12—C13	112.0 (2)
C4—C6—H61	111.5	N7—C12—C13	110.3 (2)
N7—C6—H61	106.4	C11—C12—H121	109.6
C14—C6—H61	108.5	N7—C12—H121	105.6
C6—N7—C8	105.63 (18)	C13—C12—H121	111.7
C6—N7—C12	120.1 (2)	C12—C13—H132	108.4
C8—N7—C12	112.30 (16)	C12—C13—H131	109.6
C6—N7—H71	104.2	H132—C13—H131	109.5
C8—N7—H71	105.8	C12—C13—H133	109.5
C12—N7—H71	107.6	H132—C13—H133	110.3
C3—C8—N7	103.98 (19)	H131—C13—H133	109.6
C3—C8—C9	118.7 (2)	C6—C14—O15	111.5 (2)
N7—C8—C9	109.66 (19)	C6—C14—H141	107.9
C3—C8—H81	107.1	O15—C14—H141	110.0
N7—C8—H81	106.9	C6—C14—H142	108.9
C9—C8—H81	109.8	O15—C14—H142	109.6
C8—C9—C10	109.7 (2)	H141—C14—H142	108.8
C8—C9—H92	108.9	C14—O15—H151	102.1
C10—C9—H92	109.3		

### Hydrogen-bond geometry (Å, °)

**Table:**

*D*—H···*A*	*D*—H	H···*A*	*D*···*A*	*D*—H···*A*
O5—H51···O2	0.84	2.34	2.684 (3)	105
O5—H51···O15	0.84	2.53	3.018 (3)	118
N7—H71···Br1	0.98	2.29	3.268 (2)	172
O2—H21···Br1^i^	0.82	2.55	3.364 (2)	177
O15—H151···Br1^ii^	0.84	2.39	3.211 (2)	169

Symmetry codes: (i) −*x*, *y*−1/2, −*z*+3/2; (ii) *x*, *y*−1, *z*.
